# Spontaneous Regression of Epstein-Barr Virus-Positive Diffuse Large B-cell Lymphoma in an HIV-Positive Patient: A Case Report and Literature Review

**DOI:** 10.7759/cureus.55790

**Published:** 2024-03-08

**Authors:** Evelyn Li Yuen Khaw, Wee Fu Gan, Nor Zaila Zaidan

**Affiliations:** 1 Internal Medicine, Hospital Melaka, Melaka, MYS; 2 Infectious Diseases, Hospital Melaka, Melaka, MYS

**Keywords:** diffuse large b-cell lymphoma, hiv, lymphoma, spontaneous regression, antiretroviral therapy, case report

## Abstract

Individuals infected with human immunodeficiency virus (HIV) have a greater risk of developing malignancies, including both acquired immunodeficiency syndrome (AIDS)-defining malignancies as well as many non-AIDS-defining cancers. Several factors contribute to the increased incidence of malignancies in this population such as the direct effects of HIV itself, immune deficiency, co-infection with oncogenic viruses, environmental factors, and the effects of combination antiretroviral therapy (cART). The improvement of the immune response following the introduction of cART results in a better response to conventional therapies for malignancies, including chemotherapy, radiotherapy, and surgery. Significant disparities still exist in cancer treatment for people living with HIV and afflicted with cancers compared to those without HIV, with many in the former group not receiving any cancer treatment at all. We report a rare case whereby a newly diagnosed HIV-infected patient with Epstein-Barr virus-positive diffuse large B-cell lymphoma showed spontaneous regression of the lymphoma with the introduction of cART alone without any treatment of the cancer itself. We reviewed similar cases described in the literature and examined the possible explanations for this phenomenon.

## Introduction

Since the introduction of combination antiretroviral therapy (cART), the incidence of malignancies affecting people living with human immunodeficiency virus (HIV) has declined significantly. However, acquired immunodeficiency syndrome (AIDS)-related lymphomas remain one of the most prevalent causes of morbidity and mortality in HIV-positive patients. In developed countries, non-Hodgkin lymphoma (NHL) accounts for approximately 23-30% of AIDS-related mortality [1]. This condition commonly occurs in those with an advanced stage of HIV infection, low CD4 titer, high-level viremia, and no prior exposure to cART [1-3]. AIDS-associated NHL tend to have a few distinguishable characteristics such as they tend to present at an advanced clinical stage and are of higher grade [1-3]. Extranodal involvement and presence of B symptoms are also typical with a shortened survival span as opposed to non-HIV-infected individuals [1-3]. The most common subtype of NHL in people living with HIV is diffuse large B-cell lymphoma (DLBCL), which comprises up to 50% of all lymphomas in this population group [1].

The prognosis of HIV-positive patients with NHL before the widespread use of cART was poor, with median survival ranging from 2 to 13 months despite being treated with chemotherapy [2]. Lascaux et al. [4] and Diamond et al. [5] in two separate studies estimated the median survival duration among AIDS-associated NHL patients receiving both cART and chemotherapy to be 22 months and 33 months, respectively. Since the advent of cART, survival rates for HIV-positive patients with NHL have been somewhat conflicting. A report from the Italian Cancer Registry illustrated a five-year survival rate of 25% among AIDS patients with NHL compared to 64% among HIV-negative patients with NHL from 1996 to 2005 [3]. In contrast, in a study of DLBCL patients treated with rituximab-based regimens by Coutinho et al. in the United Kingdom, HIV-positive patients had a significantly higher five-year overall survival (78% vs. 64%, p = 0.03) and disease-free survival rates (94% vs. 77%, p = 0.03) as opposed to the general population without HIV [6].

Several case reports and case series [7,8] have described spontaneous regression of lymphomas following the commencement of cART but are still extremely rare, especially in the Asian continent. We report a case of an HIV-infected patient with spontaneous regression of Epstein-Barr virus (EBV)-positive DLBCL of the colon following immune reconstitution with cART alone and reviewed six other patients based on their epidemiological, clinical, and virological features, as well as their outcomes.

## Case presentation

A 32-year-old Chinese gentleman presented with prolonged non-productive cough, shortness of breath, weight loss of 3 kg, and generalized body weakness of one-month duration. Physical examination was unremarkable except for the presence of oral thrush. HIV testing was positive with a CD4 count of 2 cells/mm^3^ and an HIV viral load of 148 copies/mL. He was treated for oral candidiasis, late latent syphilis, and *Pneumocystis jiroveci* pneumonia (PJP) which was confirmed with a sputum sample. He was discharged and planned for prompt commencement of cART after completing PJP treatment as per national guidelines.

Upon clinic review approximately two weeks later, the patient developed bilateral lower limb weakness suspicious of spinal cord pathology and was admitted for further workup. Computed tomography (CT) of the brain was unremarkable and magnetic resonance imaging (MRI) of the thoracolumbar spine showed a small L2 focal intradural extramedullary lesion. Cerebrospinal fluid (CSF) examination demonstrated a high cytomegalovirus (CMV) viral load of 1,684 IU/mL with positive fungal culture for *Sporothrix*. No biopsy of the spinal lesion was done due to its extremely small size which was deemed unsuitable for sampling after discussion with a neurologist.

Other significant history included hematochezia, multiple ulcers around the perianal region, and swollen right testis. Ultrasound imaging of the scrotum confirmed the swelling to be infective in origin, and the lesion subsequently resolved with an antimicrobial. A colonoscopy to rule out other opportunistic infections showed multiple aphthous ulcers with areas of raw bleeding from the left colon. Biopsies were taken from the mentioned lesions in the left colon while samples from the right colon were biopsied randomly and sent for histopathological examination. Intravenous amphotericin B deoxycholate and ganciclovir were initiated inpatient for the sporotrichosis and central nervous system CMV infection, which were subsequently switched to oral maintenance therapy. His condition gradually improved and he was started on cART comprising tenofovir disoproxil fumarate, emtricitabine, and efavirenz before discharge.

The histopathological examination of the biopsy samples taken from the left colon showed features of high-grade B-cell lymphoma. There were positive expression of CD20, BCL2, BCL6, Cmyc, LCA, CD45RO, CD79a, PAX5, and MUM1. The tumor cells stained negative for CD3, CD5, CD10, CD23, CD138, CD56, CD15, Cyclin D1, ALK, and CMV. EBV-encoded RNA (EBER) stain was positive with a ki67% proliferation index of >90%. A second opinion from a lymphoproliferative disorder pathologist was obtained, and it was concluded that the patient had EBV-positive DLBCL. CT staging demonstrated a few tiny mesorectal lymph nodes, subcentimeter para-aortic and aortocaval nodes, subcentimeter left supraclavicular nodes, and multiple lung nodules of varying sizes, with the largest measuring 5 mm. Otherwise, there was no focal lesion in the other organs.

**Figure 1:**
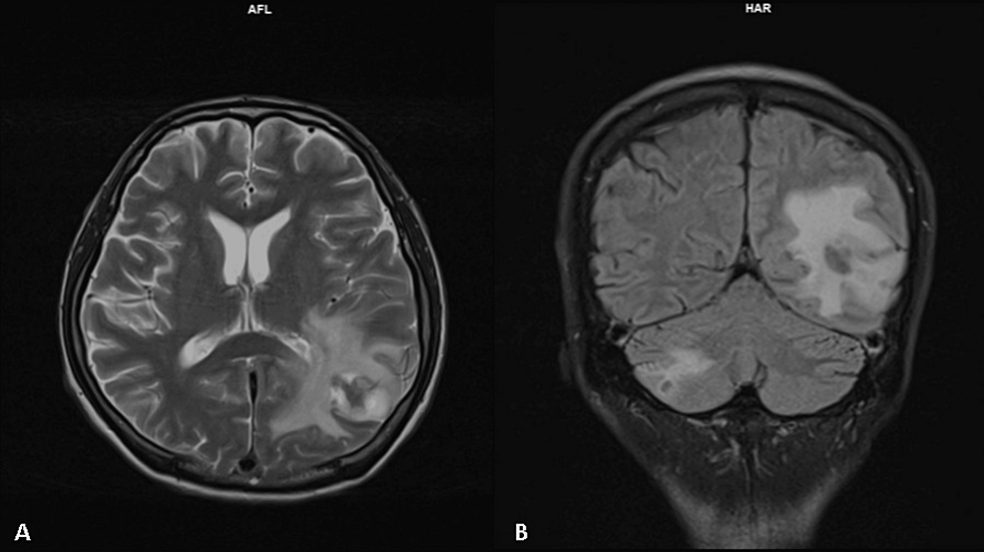
Magnetic resonance imaging of the brain suggestive of tuberculomas. Axial view (A) showing a well-defined nodular rim-enhancing white matter lesion with central non-enhancing hypointensity at the left parieto-occipital lobe measuring approximately 2.2 x 2.2 x 1.7 cm. There is marked perilesional white matter edema extending to the left temporal lobe. Minimal leptomeningeal enhancement is seen in the adjacent left cerebral hemisphere. The coronal view (B) shows another smaller lesion with similar nodular rim enhancement seen at the right cerebellar hemisphere measuring approximately 1.5 x 1.3 x 1.1 cm associated with minimal surrounding perilesional edema and adjacent leptomeningeal enhancement.

Since the diagnosis of HIV was made, the patient was admitted for the third time within two months with altered consciousness. MRI of the brain demonstrated two nodular rim-enhancing lesions in the left parieto-occipital lobe and right cerebellum associated with leptomeningeal enhancement suggestive of tuberculomas (Figure 1). CSF examination also showed high protein associated with lymphocytosis, which further strengthened our diagnosis of intracranial tuberculosis, and he was subsequently started on antituberculosis medications. CSF flow cytometry was not sent as this test was not available in our center and CSF cytology was negative for malignancy. The hematology team was consulted and he was planned for conservative management of his lymphoma due to multiple ongoing opportunistic infections and poor performance status with an Eastern Cooperative Oncology Group score of 3. No further invasive investigations such as positron emission tomography-CT scan, brain biopsy, or bone marrow biopsy were done as our patient was planned for palliative care.

**Figure 2:**
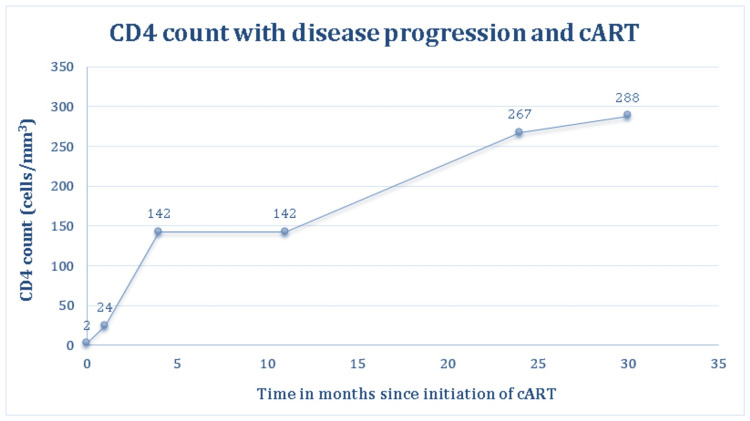
CD4 count with disease progression and cART. cART: combination antiretroviral therapy

During subsequent clinic visits, our patient had no gastrointestinal symptoms, B symptoms, and no new lymph nodes. There was marked improvement clinically as well as biochemically and he maintained compliance with the antituberculosis medications, antifungal, and cART. A repeated contrast-enhanced CT of the brain showed resolution of the brain lesions, and his neurological symptoms also resolved on antituberculosis medications alone without any chemotherapy. In addition, a colonoscopy reassessment was performed approximately a year after the initial diagnosis of his lymphoma. No luminal mass or ulcers were found on examination and the intestinal mucosa appeared healthy with no evidence of bleeding or inflammation. Biopsies were obtained from both the right and left colon, and the histopathological examination reported no evidence of malignancy. These findings suggest that our patient’s DLBCL of the colon somehow regressed spontaneously without any conventional lymphoma treatment. He continued to thrive clinically, with significant weight gain, with an improvement in appetite, and was able to perform his activities of daily living independently. He responded well to cART alone with recovery in CD4 counts to >200 cells/mm^3^, as depicted in Figure 2, and HIV viral load became suppressed three months after starting cART until the present time.

## Discussion

Spontaneous regression is a rare phenomenon, estimated to occur in 1 in 60,000 to 1 in 100,000 cases of cancer [9,10]. It can be defined as the complete or partial disappearance of a tumor without treatment or in the presence of therapy which is considered insufficient to exert a significant impact on malignant disease [9-11]. This occurrence is more commonly observed in tumors such as neuroblastomas, testicular tumors, renal cell carcinomas, melanomas, and lymphomas [9,11]. However, the data surrounding HIV patients are still scarce. Sindhu et al. [7] and Griffin et al. [8] documented a total of 12 and nine cases of HIV-associated lymphomas, respectively, which underwent rapid and complete remission after the introduction of cART alone.

In this case report, we describe a newly diagnosed HIV-positive patient with EBV-positive DLBCL of the colon who achieved remission after commencing cART only without any chemotherapy. From our literature review, we have also identified another six cases of biopsy-proven lymphomas that responded to cART alone and these are summarized in Table 1 [12-17]. Patients reported by Sindhu et al. [7] and Griffin et al. [8] have been excluded from our study cohort as well as those who received some form of conventional cancer therapy concurrently with the administration of cART.

**Table 1:** A summary of patient demographic findings and virological features in all seven cases. ^a^: EFV was switched to RAL approximately after one month. ^b^: Detected in subsequent positron emission tomography scan after the follow-up period. cART: combination antiretroviral therapy; DLBCL: diffuse large B-cell lymphoma; PBL: plasmablastic lymphoma; ALCL: anaplastic large cell lymphoma; CNS: central nervous system; CMV: cytomegalovirus; EBV: Epstein-Barr virus; HHV-8: human herpesvirus-8; TDF: tenofovir disoproxil fumarate; FTC: emtricitabine; RAL: raltegravir; EFV: efavirenz; ABC: abacavir; 3TC: lamivudine; SQV: saquinavir; RTV: ritonavir; IDV: indinavir

Case	References	Gender	Age	CD4 count at lymphoma diagnosis (cells/mm^3^)	Viral load at lymphoma diagnosis (copies/mL)	Lymphoma type	Lymphoma site	Other concurrent infections/premorbids	Oncogenic viruses link	cART regime	Follow-up	Recurrence
1	Our present case	Male	32	2	148	DLBCL	Colon	CNS tuberculosis, CNS *Sporothrix*, CNS CMV infection	EBV	TDF/FTC/RAL^a^	12 months	No
2	Koszyk-Szewczyk et. al., 2010 [12]	Male	56	288	11,6944	DLBCL	Nodal	N/A	Negative workup	Data not available	3 years	No
3	Alhatem et. al., 2019 [13]	Male	60	Data not available	Data not available	DLBCL	Liver	Hepatitis C	Data not available	Data not available	5 years	No
4	Corti et. al., 2011 [14]	Female	55	215	Data not available	PBL	Oral cavity	N/A	EBV, HHV-8	ABC/3TC/EFV	10 months	No
5	Villafañe & Corti, 2011 [15]	Male	42	<50	>500,000	B-cell	Cutaneous	N/A	Data not available	ABC/3TC/SQV/RTV	2 years	No
6	Daroit et. al., 2017 [16]	Female	66	480	Not detected	PBL	Maxilla	Hypertension, Hypothyroidism	Negative workup	ABC/3TC/EFV	12 months	Yes, bone metastasis^b^
7	Teng et. al., 2011 [17]	Male	55	200	3162	ALCL	Buccal, Maxilla, Femur	N/A	Data not available	AZT/3TC/IDV	160 months	Yes (four times), but regressed after the reinstitution of ART

Five out of seven patients were male, with a median age of 55 years at diagnosis of lymphoma (Table 1). Only one patient was on cART at lymphoma diagnosis with an undetectable HIV viral load. One patient had repeated remissions of anaplastic large-cell lymphoma following repeated recommencement of cART after multiple defaults. The rest of the patients only started cART after the diagnosis of lymphoma was confirmed. Chemotherapy was not initiated in these patients due to various reasons. Three patients refused chemotherapy, two responded to cART prior to planned chemotherapy, one had a localized lesion only and the oncologist decided on cART first and observation thereafter. Our patient had poor performance status with a multitude of ongoing opportunistic infections, hence was deemed unsuitable for chemotherapy, but was initiated on cART.

The mean CD4 count at diagnosis was 205 cells/mm^3^ whereas the median HIV viral load for five out of the seven patients was 124,050 copies/mL (range: 2 to >500,000 copies/mL) (Table 1). Data on CD4 count and HIV viral load were not available for one and two patients, respectively. All seven patients were alive and well at the last follow-up, with a median progression-free survival of 24 months (range: 10-160 months) (Table 1).

Although the exact mechanism of spontaneous regression in HIV-positive patients remains unclear, numerous hypotheses have been described over the years. HIV infection has been known to induce profound cell-mediated immune deficiency in addition to activating the immune system and inducing a chronic inflammatory state [8]. The majority of reports suggested that an infectious etiology related to the pathogenesis of the lymphoma such as EBV and human herpesvirus- 8 (HHV-8) could explain the subsequent regression upon treatment with cART [8,17]. Similarly, EBV has also been implicated in lymphomagenesis in other immunodeficiency states including post-solid organ or stem cell transplant patients [8,16]. Immune function restoration is the main focus in the treatment of EBV-associated post-transplant lymphoproliferative disorders (PTLD). Primary and reactivated EBV are controlled by CD4 and CD8 T-lymphocytes, thus treatment for PTLD involves reduction of immunosuppression, immunotherapy targeting EBV-containing lymphocytes, or administration of specific cytotoxic T-lymphocytes [8].

cART functions by suppressing HIV replication and restoring CD4 T-lymphocyte counts, working alongside cellular and humoral immune responses [8,12,16]. Therefore, it has been postulated that patients who are diagnosed with HIV at the time of lymphoma detection can respond to immune reconstitution without typical cancer treatment regimens in a comparable manner to patients with PTLD. However, only two of our seven cases reviewed had a confirmed infection with an oncogenic virus. Our present case tested positive for EBV while one plasmablastic lymphoma case was associated with both EBV and HHV-8. The relevance of an infectious cause of HIV-associated lymphomas is still debatable.

On the other hand, cART may occasionally cause significant toxicity when immune reconstitution inflammatory syndrome occurs resulting in the unmasking of malignancies or opportunistic infections [8,16]. All the cases in our cohort were diagnosed with HIV and lymphoma concurrently before the initiation of cART except for one patient who unfortunately developed the lymphoma when HIV viral load was already undetectable.

Other proposed mechanisms of spontaneous regression are also related to the immune system through action in different ways such as halting cell growth and proliferation, promoting apoptosis, and stimulating natural killer cells [8,18,19]. Some literature suggests that biopsy or ablation procedures may trigger a local healing response to resolve the tumor [16,18,19], although none was observed in our cohort. Besides that, the use of herbal medicines has been postulated to contribute to the spontaneous regression of tumors as well due to their anti-inflammatory properties [18,19]. To our knowledge, the underpinning mechanism through which HIV-associated lymphomas experience spontaneous regression is most likely due to the immune reconstitution effects of cART [7-9,12-17]. All patients had markedly increased CD4 counts at remission and significantly reduced HIV viral load, which further strengthens this theory.

## Conclusions

In summary, we described a case of spontaneous regression of EBV-positive DLBCL of the colon in an HIV-positive patient. There are very few reported cases in which people living with HIV experienced spontaneous remission in their lymphoma with the commencement of cART alone. This case highlights the positive effect of immune reconstitution and HIV disease control on lymphomagenesis. Close collaboration between HIV physicians and hematologists is essential for the management of HIV-associated lymphomas, especially as there is great variation in the behavior of these lymphomas. Immediate commencement of cART as soon as feasible with continuous assessment of fitness for chemotherapy remains the gold standard approach for HIV-associated lymphomas. Our report supports international guidelines that recommend HIV-positive patients who are either cART-naïve or non-compliant to cART to be started on antiretroviral treatment immediately.
